# Is electrophysiological study a novel predictor for permanent pacemaker implantation after transcatheter aortic valve implantation?

**DOI:** 10.55730/1300-0144.5750

**Published:** 2023-10-25

**Authors:** Zeki ÇETİNKAYA, Deniz ELÇİK, Şaban KELEŞOĞLU, Burak CESUR, Mustafa YAŞAN, Uğur KARABIYIK, Bilge BİNGÖL, Nihat KALAY, Ramazan TOPSAKAL, Aydın TUNÇAY, Ali DOĞAN, Mehmet Tuğrul İNANÇ

**Affiliations:** 1 Department of Cardiology, Elazığ Fethi Sekin City Hospital, Elazığ, Turkiye; 2 Department of Cardiology, Faculty of Medicine, Erciyes University, Kayseri, Turkiye; 3 Department of Cardiology, Kayseri State Hospital, Kayseri, Turkiye; 4 Department of Cardiology, Kastamonu Training and Research Hospital, Kastamonu, Turkiye; 5 Department of Cardiology, Niğde Training and Research Hospital, Niğde, Turkiye; 6 Department of Cardiology, Kırşehir Training and Research Hospital, Kırşehir, Turkiye; 7 Department of Cardio Vascular Surgery Faculty of Medicine, Erciyes University, Kayseri, Turkiye

**Keywords:** Transcatheter aortic valve implantation, bundle branch block, AV block, atrial-his interval

## Abstract

**Background/aim:**

Despite advancements in valve technology and increased clinical experience, complications related to conduction defects after transcatheter aortic valve implantation (TAVR) have not improved as rapidly as expected. In this study, we aimed to predict the development of complete atrioventricular (AV) block and bundle branch block during and after the TAVR procedure and to investigate any changes in the cardiac conduction system before and after the procedure using electrophysiological study.

**Materials and methods:**

A total of 30 patients who were scheduled for TAVR at our cardiovascular council were planned to be included in the study. TAVR was performed on patients at Erciyes University Medical Faculty Hospital as a single center between May 2019 and August 2020 Diagnostic electrophysiological study was performed before the TAVR procedure and after its completion. Changes in the cardiac conduction system during the preprocedure, intra-procedure, and postprocedure periods were recorded.

**Results:**

Significant increases in baseline cycle length, atrial-His (AH) interval, his-ventricular (HV) interval and atrioventricular (AV) distance were observed before and after the TAVR procedure (p = 0.039, p < 0.001, p = 0.018, p < 0.001, respectively). During the TAVR procedure, the preprocedural HV interval was longer in patients who developed AV block and bundle branch block compared to those who did not and this difference was statistically significant (p = 0.024). ROC curve analysis revealed that a TAVR preprocedure HV value >59.5 ms had 86% specificity and 75% sensitivity in detecting AV block and bundle branch block (AUC = 0.83, 95% CI: 0.664–0.996, p = 0.013). The preprocedure HV distance was 98 ± 10.55ms in the group with permanent pacemaker implantation and the mean value in the group without permanent pacemaker implantation was 66.27 ± 15.55 ms, showing a borderline significant difference (p = 0.049).

**Conclusion:**

The prolongation of HV interval in patients with AV block and bundle branch block suggests that the block predominantly occurs at the infra-hisian level. Patients with longer preprocedural HV intervals should be closely monitored for the need for permanent pacemaker implantation after the TAVR procedure.

## 1. Introduction

After the first transcatheter aortic valve replacement (TAVR) procedure was performed in humans in Rouen, France in 2002, it quickly gained popularity worldwide [1]. Initially, this procedure was performed on patients who were deemed ineligible for surgical valve replacement but later it was extended to moderate and high-risk patients [2]. Recent randomized trials have demonstrated that TAVR is either superior or noninferior to surgical aortic valve replacement (SAVR) in low-risk patients with severe aortic stenosis which represents the majority of cases [3, 4]. Therefore, the number of TAVR procedures, estimated to be 144,000 in 2019 is expected to double by 2025 [5]. With the increasing number of TAVR procedures, clinical experience is growing while complications are decreasing. However, despite the advancements in new-generation transcatheter aortic valves, there has been an increase in the incidence of permanent pacemaker requirement and conduction defects.

During the procedure, it is believed that ischemia, hematoma, or edema due to pressure on the AV node or His bundle can lead to AV block or left bundle branch block (LBBB)[6]. If the patient has preexisting right bundle branch block (RBBB), it facilitates the development of an AV complete block due to compression on the left bundle branch. Predictors of permanent pacemaker implantation after TAVR include preexisting right bundle branch block, postprocedural left bundle branch block, small left ventricular outflow tract (LVOT) diameter, deep valve implantation, excessive balloon dilation, use of self-expandable valves, and LVOT calcification [7]. In addition, first-degree block and left anterior hemiblock on the electrocardiogram were observed as preprocedural permanent pacemaker predictors [8]. However, the role of electro-physiological parameters in predicting these outcomes has not been fully elucidated. In our study, we aimed to explore the relationship between preprocedural and postprocedural electro-physiological parameters and the development of AV complete block, bundle branch block, and the need for permanent pacemaker implantation during or after TAVR.

## 2. Materials and methods

### 2.1. Study population

This study included 30 patients between the ages of 61 and 90 who had symptomatic severe aortic stenosis were at high surgical risk and had a life expectancy of over one year. The study was conducted prospectively at Erciyes University Faculty of Medicine hospital between May 2019 and August 2020. However, a total of 7 patients were excluded from the study: 4 due to atrial fibrillation, 2 who refused the procedure, and 1 who had a permanent pacemaker.

Ethical approval was obtained from the Clinical Research Ethics Committee (decision no: 2019/301). They were included in the study after a signed informed consent form and a written consent form stating that they would participate in the study were obtained from the patients and their relatives (after the informed consent form was submitted).

### 2.2 Electrophysiological study

Patients using antiarrhythmic drugs such as B-blockers due to ischemic heart disease and congestive heart failure were discontinued three days before the procedure. We did not have any patients using antiarrhythmic drugs other than B blockers. An electrocardiogram (ECG) was taken before and after the TAVR procedure. ECG recording was taken during the procedure.

Immediately before TAVR and after the TAVR procedure, the patient was taken to the electrophysiology lab for a hybrid angiography procedure. Using a stimulator device (EP-WorkMate™, St. Jude), basic parameters such as AH interval, HV interval, basal cycle length, AV interval, AV Wenckebach point, SNRT, and cSNRT were examined. Due to the risk of arrhythmia induction, measurements of atrial and ventricular refractory periods were not performed. Surface electrograms were continuously monitored and recorded throughout the entire procedure for all patients.

### 2.3 TAVR procedure

Multidetector computed tomography (MDCT), transthoracic echocardiography (TTE) and ECG were performed before the procedure. MDCT measurements were performed by a radiologist and a cardiologist (F.Y and R.T) using the 3 mensio Structural Heart 9.1 SP3 program (manufacturer name: PIE Medical Imaging/manufacturer country: The Netherlands) by retrospectively scanning from the ExtremePacs Client program (manufacturer name:ExtremePacs/manufacturer country: Türkiye) of our hospital’s image recording system and saving to external memory. Iliac artery diameter, structure and aortic valve measurements were performed. Afterwards, the patients were operated under local anesthesia. Arterial access was established, and aortic valvuloplasty and TAVR were performed using St. Jude Portico valves (manufacturer name: Abott/manufacturer country: USA).

### 2.4 Statistical analysis

Statistical analysis was performed using the SPSS program (version 21.0, IBM Company, SPSS Inc.). The normal distribution of the data was evaluated using histograms, Q-Q plots and the Shapiro-Wilk test. When comparing two groups with normal distribution, independent two-sample t-tests were used, while the Mann-Whitney U test was used for groups with nonnormal distribution. Dependent variables with parametric distribution were analyzed using the Dependent Two-Sample t-test and the Wilcoxon test was used for variables with nonparametric distribution. The Pearson’s χ2 test was used for categorical variables. When comparing electrophysiological parameters with arrhythmia types observed on ECG, One-Way ANOVA with post hoc Tukey test was used for parametric tests with homogeneous distribution and the Kruskal-Wallis test was used for nonparametric tests. ROC curve analysis was used to determine the sensitivity and specificity of the preprocedural HV interval in predicting AV block and bundle branch block development.

## 3. Results

A total of 23 patients were included in the study, with 12 males and 11 females. The mean age of the participants was 77 (range: 61–90) years. Six patients had a history of coronary artery bypass graft (CABG) surgery and 10 patients had moderate to advanced chronic obstructive pulmonary disease. Four patients had chronic kidney failure and were under hemodialysis. Three patients had a history of cancer but had a life expectancy of more than one year. The average EuroSCORE II of the patients was 12.79 ± 7.34, indicating a high surgical risk (Table 1).

Electrophysiological basic parameters were measured before and after the TAVR procedure. The baseline cycle length was 766 (range: 647–864) ms before the procedure and 790 (range: 689–879.5) ms after the procedure, showing a statistically significant difference (p = 0.039). The AH interval was 77 (range: 65–90) ms before the procedure and 95 (range: 87.5–106) ms after the procedure, which was statistically significant (p < 0.001). The HV interval was 64 (range: 55–78) ms before the procedure and 83 (range: 56.5–98) ms after the procedure, with a significant difference (p = 0.018). The AV interval was 160 (range: 135.5–190) ms before the procedure and 188 (range: 159–221) ms after the procedure, which was statistically significant (p < 0.001) (Table 2).

Three patients had preexisting left bundle branch block (LBBB) before the procedure. Within the first 24 h, 11 patients developed LBBB, and at the 3-month follow-up, 9 patients still had LBBB. Six (26%) patients developed new and persistent LBBB. Three patients had preexisting right bundle branch block (RBBB) and all of them developed complete atrioventricular (AV) block requiring permanent pacemaker implantation. During the procedure, ventricular tachycardia occurred in one patient and VT ablation was performed after TAVR (Table 3).

The preprocedural HV interval during TAVR was 54.85 ± 5.8ms in the group without AV block or bundle branch block, 75.8 ± 46.2ms in the group with AV block and 68 ± 16.1 ms in the group with bundle branch block. There was a statistically significant difference between the group without AV block or bundle branch block and the group with AV block (p: 0.03). However, there was no significant difference between the group with bundle branch block and the group without AV block or bundle branch block. Other electrocardiographic changes during the procedure did not show a statistically significant difference compared to baseline measurements, except for HV interval (Table 4).

**Figure 1:**
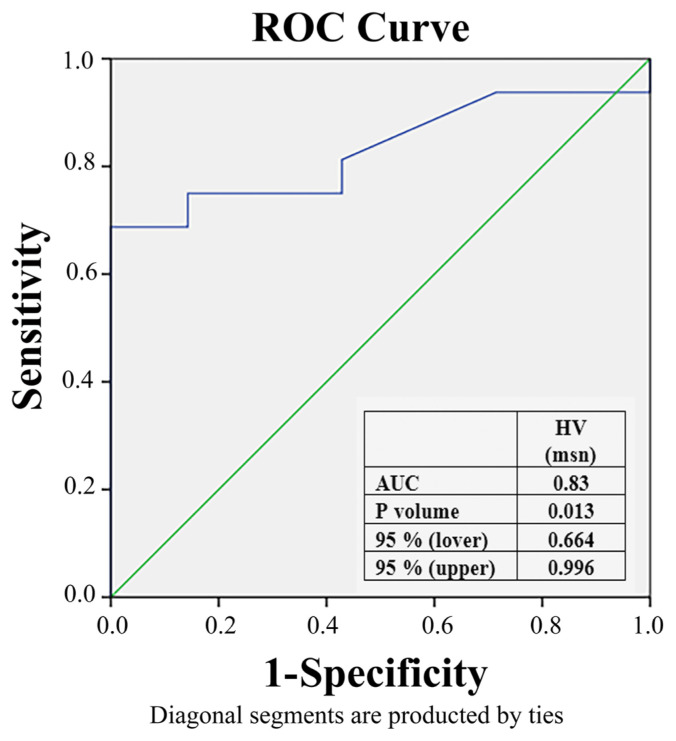
The effect of H-V interval on pacing.

During the procedure, a HV interval longer than 59.5 ms showed a sensitivity of 86% and specificity of 75% for predicting the development of AV block or bundle branch block. (AUC = 0.83, 95% CI: 0.664–0.996, p = 0.013) (Figure 1).

In the group that required permanent pacemaker implantation, the baseline cycle length was 1080 ± 129.532 ms, while it was 764.13 ± 149.332 ms in the group without pacemaker implantation, which was marginally significant (p = 0.047). The pre-procedural HV interval was 98 ± 10.55ms in the pacemaker implantation group and 66.27 ± 15.55 ms in the group without pacemaker implantation, which was marginally significant (p = 0.049). There was no significant difference in other preprocedural electrophysiological parameters based on pacemaker implantation (Table 5).

## 4. Discussion

With the increasing clinical experience and advancements in valve technologies, complications associated with TAVR have been decreasing. However, despite improvements in device technology and operator experience, the improvement in conduction abnormalities is not as rapid as expected and the incidence of new-onset LBBB and permanent pacemaker implantation has been found to be higher with some new-generation TAVR devices [9]. The development of LBBB and the need for permanent pacemaker implantation increase morbidity and mortality. Prolonged hospitalization and the cost of permanent pacemaker implantation also contribute to increased healthcare costs.

In the Partner 3 trial, TAVR using self-expanding valves was found to be noninferior to surgery in terms of mortality, stroke, and hospitalization in patients with low surgical risk [3]. The incidence of permanent pacemaker implantation was 19.4% in the TAVR group and 6.7% in the surgical group. In the Partner B trial, which focused on TAVR for high-risk aortic stenosis, the rate of permanent pacemaker implantation within the first month was 21.6%. In our study, we included patients with high surgical risk for aortic stenosis, and the rate of permanent pacemaker implantation was determined to be 4.3. Compared to the Partner 3 and Partner B trials, the rate of permanent pacemaker implantation was lower in our study.

Of the 23 patients in the study, a total of 6 patients, 3 RBBB and 3 LBBB had conduction defects before the procedure (26%), which was similar to the incidence in previous studies. The incidence varies between 12% and 35% in studies [9–11]. In a retrospective study by Becker et al., the right bundle branch block before TAVR was among the predictors of permanent pacemaker implantation after the procedure. In this study, 11 patients had RBBB and only 5 (45.4%) had a permanent pacemaker implanted [9]. In our study, RBBB was present in three patients before the procedure. A permanent pacemaker was implanted in only one of three patients (33.3%). One developed a complete AV block during the procedure. After the procedure, 60 mg methylprednisolone was administered. In the first 24 h, AV was out of the block. The other patient with RBBB did not develop any conduction defect. It is generally thought that an AV complete block will develop because the valve is damaged due to compression of the left bundle of His bundle and the patient initially had RBBB [10].

Post-procedure permanent pacemaker implantation rates for the Core Valve system range from 18%–35% [11–15]. These rates were lower for Edward-Sapiens caps [16]. These wide differences between studies may be due to fundamental differences between patients or more likely, inconsistencies in the indication for permanent pacing. In a meta-analysis of 41 studies including 11,210 patients who underwent TAVR and 17% had permanent pacemaker implantation, male gender, left anterior hemiblock, RBBB, grade 1 AV block and those with AV block during the procedure were identified as preprocedural predictors of postprocedural permanent pacemaker implantation [17]. In this meta-analysis, it was found that there were 2.5 times more permanent pacemaker implantations with Medtronic Core Valve and Edwards SAPIEN Valve compared to other valves. In our study, only one patient (4.3%) out of 23 patients, a male, required permanent pacemaker implantation. The rate of permanent pacemaker implantation in our study was much lower (4.3%) compared to this metaanalysis. However, the difference between our study and this metaanalysis is that we used St. Jude Portico and Medtronic Core Valve Evolute R valves. Our sample size is smaller compared to this metaanalysis. RBBB was present in 3 patients before the procedure. 2 were implanted with Medtronic Core Valve Evolute and the other with St. Jude Portico valve. A permanent pacemaker was implanted only in the patient with Medtronic Core Valve Evolut valve. There was no statistically significant difference between the valves (p = 0.074). It is thought that there will be a statistically significant difference when the sample size is increased. Furthermore, in our study, if the HV interval was above 59.5 ms before the TAVR procedure, it was observed that there was an 86% specificity and 75% sensitivity in predicting the development of AV block or bundle branch block during the procedure (p = 0.013) (Figure 1). One of the preprocedural predictors for permanent pacemaker implantation after the TAVR procedure can be the HV interval being above 59.5 ms.

**Figure 2:**
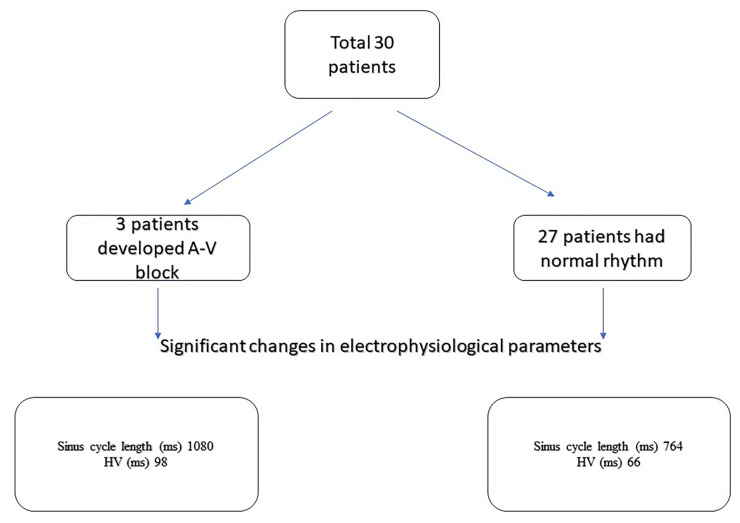
Study diagram.

In our study, AH, HV, AV duration and basal cycle length were statistically significantly increased after the procedure compared to before the procedure. Rubin et al. investigated the effect on AV block by performing superficial ECG and intracardiac measurements before and after implantation of the 3rd generation core valve in 18 patients [18]. The AH interval before the procedure was 97 (70–123) ms, and after the procedure, it increased to 115 (96–135) ms, showing a statistically significant increase (p = 0.0021). The HV interval before the procedure was 52 (42–55) ms and after the procedure, it increased to 60 (50–70) ms, which was also statistically significantly increased (p = 0.002). The cSNRT interval before the procedure was 200 (138–285) ms and after the procedure, it was 210 (148–290) ms, with no statistically significant difference observed (Figure 2). The parameters that showed statistically significant increases in electrophyiological basic parameters were similar to our study.

During the TAVR procedure, it was observed that there was a statistically significant increase in the HV interval in the group with AV block and bundle branch block compared to the group without AV block and bundle branch block (p = 0.03). However, there was no significant difference in the AH interval between the groups. These findings suggest that the AV block is likely to occur at the infra-Hisian level.

In the group without permanent pacemaker implantation, the HV interval was 66.27 ± 15.55 ms, while in the group with permanent pacemaker implantation, it was 98 ms, which was marginally significant (p = 0.059). With an increased sample size, there may be a significant change in this value and it can be speculated that in the group with pacemaker implantation, the block may develop at the infra-Hisian level due to a direct effect on the infra-Hisian conduction system, possibly caused by direct compression of the lower region of the prosthesis on the basal part of the ventricle.

The most important limitation of this study is the small number of cases and the single centre. The results may be clearer with larger studies. In addition, since postprocedure follow-ups could not be given, the need for subsequent pacing and the factors affecting this can be evaluated in other studies.

In conclusion, our study showed a significant increase in the HV interval in patients who developed AV block during the procedure. Therefore, attention should be paid to AV block and pacemaker requirements in such patients. This increase indicates that the block is likely to be at the infra-hisian level. There was no significant difference in preprocedural electrophysiological parameters between patients with and without permanent pacemaker implantation. However, with a larger sample size, it is expected that a long preprocedural HV interval may be included among the indications for permanent pacemaker implantation after TAVR. This value can be further supported by increasing the sample size.

## Figures and Tables

**Table 1:** Baseline all patients.

	All Patients (n: 23)	Male (n: 12)	Female (n: 11)	p-values
Age	77 (61–90)	76 (61–90)	77(65–83)	0.62
Hypertension	18 (78.3)	8 (66.7)	10 (90.9)	0.317
Diabetesmellitus	14 (60.9)	6 (50)	8 (72.7)	0.4
CAD	16 (69.6)	9 (75)	7 (63.6)	0.66
CABG	6 (26.1)	4 (33.3)	2 (18.2)	0.64
COPD	1 0 (43.5)	5 (41.7)	5 (45.5)	1
Smoker	6 (26.1)	6 (50)	0	0.008
Renal Failure	4 (17.4)	3 (25)	1 (9.1)	0.59
PAD	1 (4.3)	1 (8.3)	0	0.328
Cancer	3 (13.6)	2 (18.2)	1 (9.1)	0.534
Cheast pain	12 (52.2)	5 (41.7)	7 (63.6)	0.292
Syncope	4 (17.4)	3 (25)	1 (9.1)	0.59
Dizziness	15 (65.2)	7 (58.3)	8 (72.7)	0.66
Shortness of breath	23 (100)	12 (100)	11 (100)	1
NYHA class 2	1 (4.3)	0	1 (9.1)	0.537
NYHA class 3	14 (60.9)	8 (66.7)	6 (54.5)	
NYHA class 4	8 (25)	4 (33.3)	4 (36.4)	
Aortic peak gradiyent	67.5 (61.5–83)	69.2 ± 16.3	70.2 ± 13.3	0.53
Aorticmean gradiyent gradiyent	45.3(41.9–50)	44.1 ± 11.3	45.4 ± 7.2	0.27
AorticValvearea(cm^2^)	0.74 ± 0.23	0.91 ± 0.34	0.84 ± 0.35	0.94
BMI, kg/m^2^	29.94 ± 4.9	26.96 ± 2.12	33.2 ± 5.23	0.001
Euro score 2	12.79 ± 7.34	14.8 ± 9.1	10.55 ± 4	0.166
STS-PROM	7.63 ± 3.8	6.12 ± 2.85	9.27 ± 4.3	0.056
Medical treatment				
B-blocker	8 (34.8)	5 (41.7)	3 (27.3)	0.46
Calcium antagonistic	2(8.6)	1 (8.3)	1 (9.1)	0.94
ACE-inhibithor	11 (47.8)	6 (50)	5 (45.5)	0.82
ARB	5 (21.7)	3 (25)	2 (18.2)	0.69
Statin	12 (52.2)	6(50)	6 (54.5)	0.82
Antiaggregant	16 (69.6)	9 (75)	7 (63.6)	0.66

ACE: Angiotensin-converting Enzyme, ARB: Angiotensin Receptor Blocker, BMI: Body Mass Index, CAD: Coronary Artery Disease, PAD: Peripheral Arterial Disease, STS-PROM: The Society of Thoracic Surgeons Predicted Risk of Mortality.

**Table 2:** Comparison of electrophysiological parameters before and after TAVR.

Electrophysiological basic parameters	Before TAVR	After TAVR	p-values
Sinus cycle length (ms)	766 (647–864)	790 (689–879.5)	0.039^*^
SNRT (ms)	929 (867–1081)	718 (936–1076)	0.121
Csnrt (ms)	194 (161.5–250)	230 (148–315)	0.632
AH (ms)	77 (65–90)	95 (87.5–106)	<0.001^*^
HV (ms)	64 (55–78)	83 (56.5–98)	0.018
AV (ms)	160 (135.5–190)	188 (159–221)	<0.001^*^
AV Wenkebach (ms)	340 (305–375)	350 (315–405)	0.079

AH: Atrial His, Av: Atrio-Ventricular, cSNRT(msn): Corrected Sinus Node Recovery Time, HV: His-ventricular, SNRT: Sinus Node Recovery Time, TAVR: Transcatheter Aortic Valve Replacement

**Table 3:** ECG parameters during TAVR.

	Before TAVR	During TAVR	First 24 h after TAVR	Discharge
Narrow Qrs	17 (73.9)	4 (17.3)	5 (21.7)	11 (47.8)
Lbbb	3 (13)	1 0 (43.4)	11 (47.8)	9 (39.1)
Rbbb	3 (13)	1 (4.3)	1 (4.3)	0
Av complete block Pacemaker	0	6 (26)	5 (21.7)	1 (4.3)
VT	0	1 (4.3)		
Asistol/Exitus	0	1 (4.3)		

Av: Atrio-Ventricular, LBBB: Left Bundle Branch Block, RBBB: Right Bundle Branch Block VT: Ventricular Tachycardia, TAVR: Transcatheter Aortic Valve Replacement

**Table 4:** Comparison of basal electrophysiological properties and ECG changes during TAVR procedure.

Electrophysiological basic parameters	Patients who do not develop Av blockor BBB during TAVR procedure (n: 7)	Patients who develop Av blok during TAVR procedure (n: 7)	Patients who develop BBB TAVR procedure (n: 9)	p-values
Sinuscycle length (ms)	735.8 ± 187.5	804.4 ± 151.3	789.8 ± 156.7	0.71
SNRT (ms)	948.14 ± 235.7	1060.33 ± 166.14	966 ± 153.3	0.72
cSNRT(ms)	176.5 (163.25–243.2)	167 (109–376)	232 (143–305)	0.81
AH (ms)	74.7 ± 11.3	97.7 ± 34.3	75.3 ± 18	0.11
HV (ms)	54.85 ± 5.8^a^	75.8 ± 46.2^b^	68 ± 16.1^ab^	0.03
AV(ms)	148.4 ± 19.5	183.4 ± 44.3	166.1 ± 36.9	0.2
AV Wenkebach(ms)	360 (320–370)	330 (310–410)	330 (295–370)	0.63

AH: Atrial His, Av: Atrio-Ventricular, BBB: Bundle Branch Block, cSNRT(msn): Corrected Sinus Node Recovery Time, HV: His-ventricular, SNRT: Sinus Node Recovery Time, TAVR: Transcatheter Aortic Valve Replacement

**Table 5:** Preoperative electrophysiological parameters of groups with and without permanent pacemaker.

Electrophysiologi-cal basic parameters	Permanent pacemaker implaneted group (n: 3)	Patients who do not need permanent pacemaker (n: 20)	p-values
Sinuscycle length (ms)	1080 ± 129.532	764.13 ± 149.332	0.047
SNRT (ms)	1247 ± 119.32	976.47 ± 175.21	0.147
Csnrt (ms)	167(152–243)	194 (154–307)	0.52
AH (ms)	106 (67–118)	74.5 (65–90)	0.22
HV (ms)	98 ± 10.55	66.27 ± 15.55	0.049
AV(ms)	198 ± 29.4	164.55 ± 36.6	0.382
AV Wenkebach (ms)	410.1 ± 39.532	341.36 ± 47.539	0.173

AH: Atrial His Av: Atrio-Ventricular BBB: Bundle Branch Blockc SNRT(msn): Corrected Sinus Node Recovery Time SNRT: Sinus Node Recovery Time
